# *Drosophila melanogaster* Hsp22: a mitochondrial small heat shock protein influencing the aging process

**DOI:** 10.3389/fgene.2015.00103

**Published:** 2015-03-16

**Authors:** Geneviève Morrow, Robert M. Tanguay

**Affiliations:** Laboratoire de Génétique Cellulaire et Développementale, Département de Biologie Moléculaire, Biochimie Médicale et Pathologie, Institut de Biologie Intégrative et des Systémes and PROTEO, Université LavalQuébec, QC, Canada

**Keywords:** Hsp22, *Drosophila melanogaster*, aging, mitochondria, mitochondrial unfolding protein response

## Abstract

Mitochondria are involved in many key cellular processes and therefore need to rely on good protein quality control (PQC). Three types of mechanisms are in place to insure mitochondrial protein integrity: reactive oxygen species scavenging by anti-oxidant enzymes, protein folding/degradation by molecular chaperones and proteases and clearance of defective mitochondria by mitophagy. *Drosophila melanogaster* Hsp22 is part of the molecular chaperone axis of the PQC and is characterized by its intra-mitochondrial localization and preferential expression during aging. As a stress biomarker, the level of its expression during aging has been shown to partially predict the remaining lifespan of flies. Since over-expression of this small heat shock protein increases lifespan and resistance to stress, Hsp22 most likely has a positive effect on mitochondrial integrity. Accordingly, Hsp22 has recently been implicated in the mitochondrial unfolding protein response of flies. This review will summarize the key findings on *D. melanogaster* Hsp22 and emphasis on its links with the aging process.

## Introduction

Aging is associated with a decline in protein homeostasis (proteostasis) that leads to the accumulation of deleterious protein damages. Mitochondrial proteins are particularly prone to accumulate damages due in part to the close proximity of the ETC. To circumvent deleterious accumulation of protein damages, three types of mechanisms are involved in mitochondrial PQC (reviewed in Kotiadis et al., 2014). The first line of defense comprises both anti-oxidants enzymes that scavenge ROS produced as by-product of the ETC and molecular chaperones and proteases that insure protein folding or degradation of damaged proteins (reviewed in Bozaykut et al., 2014). The third mechanism of mitochondrial PQC involves the clearance of highly damaged mitochondria through mitophagy (reviewed in Osellame and Duchen, 2014; Scheibye-Knudsen et al., 2015).

Heat shock proteins are molecular chaperones found in all organisms. They are subdivided in distinct families based on their molecular weight and sequence homology: HSP110, HSP90, HSP70, HSP60, HSP40, and sHSP. Each HSP family has specific functions that have been the subject of different reviews (Vos et al., 2008; Priya et al., 2013; Ryabova et al., 2013; Saibil, 2013; Karagoz and Rudiger, 2015). The sHSPs are characterized by the presence of the alpha-crystallin domain and are at the crossroad between two main process namely protein folding and degradation. Indeed, most sHSPs have the ability to prevent protein aggregation and to maintain their client in a refoldable state hence preventing them to form deleterious interactions (reviewed in Haslbeck et al., 2005; McHaourab et al., 2009; Fu, 2014; Treweek et al., 2015). Additionally, some sHSPs are involved in protein degradation via the proteasome and autophagy (Goldbaum et al., 2009; Bissonnette et al., 2010; Acunzo et al., 2012).

In *Drosophila melanogaster* there are 12 members of the sHSP family that have different chaperone ability, distinctive intracellular localization and cell- and stage-specific pattern of expression (Michaud et al., 2002; Vos, 2009; Morrow and Tanguay, 2015). Almost all of them are stress-inducible, but only seven have been shown to be up-regulated during aging (CG14207, l(2)efl, Hsp67Bc, Hsp22, Hsp23, Hsp26, and Hsp27; King and Tower, 1999; Zou et al., 2000; Landis et al., 2004, 2012; Wang et al., 2005; Girardot et al., 2006; Tanguay and Morrow, 2008; Yang and Tower, 2009). Among these sHSPs, the link between Hsp22 and aging is particularly interesting due to its peculiar mitochondrial matrix localization (Morrow et al., 2000) and given the central role of mitochondria in the aging process (Hill and Van Remmen, 2014; Ziegler et al., 2014). Mitochondria are involved in different metabolic and signaling pathways (ATP production, amino acid catabolism, fatty acid β-oxidation, apoptosis among others) and are in constant communication with the nucleus to adjust to metabolic demand (Haynes et al., 2007; Haynes and Ron, 2010; Runkel et al., 2014). While ROS produced by mitochondria have been at the center of the free radical theory of aging (Harman, 1956), recent reports are now showing that increased ROS production is not always harmful and can even promote longevity (Van Raamsdonk and Hekimi, 2009, 2012; Yee et al., 2014). In recent years, multiple factors have been shown to contribute to aging by favoring accumulation of dysfunctional mitochondria such as impairment of mitochondria-to-nucleus signaling, changes in mitochondrial dynamics (fusion/fission) and clonal amplification of mitochondrial DNA mutations (Bereiter-Hahn, 2014; Hepple, 2014; Ziegler et al., 2014). A failure to maintain mitochondrial homeostasis and integrity is therefore associated with aging (Bratic and Larsson, 2013; Bohovych et al., 2014) and accordingly, the maintenance of mitochondrial stress response has gained recognition as a potential pro-longevity mechanism (Hill and Van Remmen, 2014; Scheibye-Knudsen et al., 2015).

## Hsp22 is Preferentially Up-Regulated During Aging

As a member of the sHSP family, Hsp22 is readily up-regulated by a variety of different stresses (Colinet et al., 2010; Hirano et al., 2012; Landis et al., 2012; Morrow and Tanguay, 2015) but its developmental expression pattern is tightly regulated. Indeed, during development its expression is restricted to the metamorphosis of larvae to pupae (Michaud et al., 2002). However, during adulthood Hsp22 is the most up-regulated sHSP, the induction of its mRNA reaching up to 60% in the head of 30 days-old flies comparatively to 6 days-old flies (King and Tower, 1999; Yang and Tower, 2009; Landis et al., 2012). Since *hsp22* mRNA is post-transcriptionally regulated, the protein was only detected starting at 40 days of age in these flies (King and Tower, 1999), and the resulting increase was of ≥150%.

Interestingly, fly strains genetically selected for their increased longevity display increased *hsp22* mRNA at the beginning of adulthood comparatively to short-lived strains (Kurapati et al., 2000; Zhao et al., 2005b). These flies were also more resistant to heat-shock and were shown to have a quicker heat-shock response than short-lived flies (Zhao et al., 2005b) suggesting a beneficial role of Hsp22 during aging (Kurapati et al., 2000; Zhao et al., 2005b). This was further confirmed by over-expression and down-regulation studies (see Hsp22 Over-Expression Increases Longevity and Resistance to Stress and Absence of Hsp22 Expression Decreases Lifespan and Resistance to Stress, Morrow et al., 2004a,b). This positive correlation between the *hsp22* mRNA level and lifespan likely indicates a more effective stress response and is consistent with a report showing a positive correlation between the level of induction of a *shsp* reporter in response to stress and the remaining lifespan in *Caenorhabditis elegans* (Rea et al., 2005; Yang and Tower, 2009).

### Hsp22 Expression Partially Predicts the Remaining Lifespan of Flies

Due to its stress-inducibility (Colinet et al., 2010; Hirano et al., 2012; Landis et al., 2012; Morrow and Tanguay, 2015) and to the fact that the onset of Hsp22 protein induction is near the beginning of the period of rapid death in the fly population (King and Tower, 1999), the ability of Hsp22 to be an aging biomarker was investigated using transgenic flies expressing the green fluorescent protein (GFP) driven by an *hsp22* promoter (*hsp22*-GFP; Yang and Tower, 2009). It was shown that in a given strain, flies that were robustly expressing the *hsp22-*GFP transgene at younger adult age than their counterpart tended to die sooner. In this case, the abnormal level of *hsp22-*GFP transgene expression would be indicative of the high level of stress experienced by a given individual and would represent that particular individual’s susceptibility to stress and failing homeostasis and as such could serve as a stress biomarker announcing imminent mortality (Yang and Tower, 2009). While this may seem contradictory to other studies on the beneficial effects of Hsp22 on longevity (Kurapati et al., 2000; Morrow et al., 2004b; Zhao et al., 2005b), it may only represent the importance to express Hsp22 early in development to observe its effect on lifespan. Unfortunately no data on mRNA/protein level of *hsp22* are available for the long-lived strains harboring increased levels of *hsp22* mRNA early in development (see Hsp22 is Preferentially Up-Regulated During Aging, Kurapati et al., 2000; Zhao et al., 2005b). However, studies using over-expression of Hsp22 have clearly shown that it must be expressed before 4 days of age to confer an increased longevity (Bhole et al., 2004; Morrow et al., 2004b). Moreover, the sensitivity of GFP detection technique may not favor the detection of weak/transient *hsp22*-GFP expression and therefore emphasis on more robust expression. In the same way, Hsp22 protein expression driven in adult motorneurons was not observable by western blots of whole fly homogenate but was still able to mediate lifespan increase (Morrow et al., 2004b). Together these data suggest that the cell-types in which Hsp22 is expressed and the timing of its expression are important factors in its beneficial effect on aging and that robust expression of Hsp22 at the whole organism level may reflects intensive stress and failing homeostasis.

## Hsp22 Expression is Modulated by Factors Influencing Longevity

In the course of understanding the aging process, different proteins, and pathways have been shown to influence lifespan. Interestingly, in some cases the modulation of Hsp22 expression was also reported.

### dFoxo as a Regulator of Hsp22 Expression

The up-regulation of Jun-*N*-terminal Kinase pathway and the down-regulation of the insulin/IGF pathway converge to the transcription factor Foxo to increase lifespan and stress tolerance (Tatar et al., 2001; Wang et al., 2003, 2005; Accili and Arden, 2004; Giannakou and Partridge, 2007). In *D. melanogaster*, dFoxo has been shown to regulate the expression of Hsp22 together with Hsp23, CG14207, l(2)efl, Hsp70, Hsp40, Hsp90, and Hop (Wang et al., 2005; Harvey et al., 2008; Hull-Thompson et al., 2009; Demontis and Perrimon, 2010). Hsp22, Hsp23, and l(2)efl increase longevity upon over-expression and it was therefore proposed that they are, at least in part, involved in the lifespan extension mediated by dFoxo (Morrow et al., 2004b; Wang et al., 2005; Tanguay and Morrow, 2008). Accordingly, it was shown that dFoxo null flies have a reduced lifespan as well as a reduced age-induced expression of l(2)efl (Shen and Tower, 2010) and Hsp22 (Morrow and Tanguay, 2015).

### Hsp22 Expression is Coordinated with the Life Promoting Protein dDnmt2

In flies, dDnmt2 is the only DNA methyl transferase known up to now. Contradictory with its name dDnmt2 has a relatively poor DNA methyl transferase activity, but it has, however, a rather robust tRNA methyl transferase activity (Schaefer and Lyko, 2010; Schaefer et al., 2010). One of the functions of dDnmt2 is to protect stress-induced cleavage of tRNA in stress granules (Schaefer et al., 2010) and it would also be a life determination gene since it increases lifespan and resistance to oxidative stress upon over-expression and decreases *Drosophila* lifespan when down-regulated (Lin et al., 2005; Schaefer et al., 2010). Interestingly, only Hsp22, Hsp23, and Hsp26 (and no other life promoting genes such as Inr, chico, metuselah, and SOD) were shown to be expressed similarly to dDnmt2 (i.e., up-regulated when dDnmt2 is over-expressed or down-regulated when dDnmt2 expression is decreased) suggesting that the lifespan determination of dDnmt2 is interconnected with sHSP expression (Lin et al., 2005).

### Hsp22 Expression is Influenced by Histone Methylation and Acetylation

Histone post-translational modifications are known to control gene transcription. Among the enzymes regulating modifications of histones, the histone demethylase KDM4A has been suggested to regulate longevity gene expression. Indeed, the depletion of KDM4A has been shown to induce cellular senescence in normal fibroblasts (Mallette and Richard, 2012) and to decrease lifespan in flies (Lorbeck et al., 2010). Interestingly, the most down-regulated gene in short-lived KDM4A flies was *hsp22* (Lorbeck et al., 2010). A link between Hsp22 expression and histone acetylation has also been observed in flies. Indeed, inhibition of histone deacetylase by trichostatin and sodium butyrate was shown to increase lifespan and promote *hsp22* and *hsp70* expression (Zhao et al., 2005b). In this case, the binding of hyperacetylated histone H3 at both promoters was shown to increase accessibility of HSEs to the heat shock factor (Zhao et al., 2005a).

## Hsp22 has a Protective Role During Lifespan

The beneficial role of Hsp22 during aging was shown by over-expression and down-regulation studies in flies and was also demonstrated in human cells.

### Hsp22 Over-Expression Increases Longevity and Resistance to Stress

Using the Gal4-UAS system, it was shown that over-expression of Hsp22 either ubiquitously with the actin driver or in motorneurons with the D42 driver increases resistance to heat and oxidative stresses and longevity by up to 30% (Morrow et al., 2004b). Moreover, flies over-expressing the sHSP maintained their locomotor activity for a longer time suggesting that Hsp22 over-expression increases the health-span (Morrow et al., 2004b). While the beneficial effect of Hsp22 over-expression on lifespan is clear in this system, the timing of its expression is very important. Indeed, over-expressing the sHSP at the beginning of adulthood instead of the beginning of embryogenesis did not result in any increase of longevity (Bhole et al., 2004).

### Absence of Hsp22 Expression Decreases Lifespan and Resistance to Stress

The three HSEs of the *hsp22* promoter are required for the age-induced expression of Hsp22 (King and Tower, 1999). Accordingly, flies that carry a p-element insertion in the *hsp22* promoter (in between the HSEs) lack the sHSP expression during aging and consequently have a decreased longevity and resistance to stress (Morrow et al., 2004a).

### Hsp22 Increases Population Doubling in Human Fibroblasts

Ten sHSPs are found in humans and up to now none has been found to reside constitutively inside the mitochondria. Interestingly, over-expression of *D. melanogaster* Hsp22 in primary human fibroblasts extended their lifespan from 58 population doublings to 84 population doublings and this was accompanied by a lower level of the senescence associated β-galactosidase marker (Wadhwa et al., 2010). While it is clear that Hsp22 was functionally active in human cells, its expression was also shown to increase malignant properties of human cancer cell lines (Wadhwa et al., 2010). In mammals, sHSPs are up-regulated in many different cancer cell types and are often linked to bad prognoses (Boncoraglio et al., 2012; Kampinga and Garrido, 2012). The exact mechanisms by which Hsp22 operate in human cells has not been investigated deeply. However, p53 was shown to co-immunoprecipitate with Hsp22 and accordingly, p53 was found in the mitochondria of Hsp22 over-expressing cells (Wadhwa et al., 2010).

## Hsp22 Over-Expression Triggers Changes in Gene Transcription

Consistent with the extent of Hsp22 beneficial effect at the organismal level, Hsp22 over-expression was shown to alter gene transcription. Indeed, transcripts from protein involved in multiple functions were shown to be expressed differently in Hsp22 over-expressing flies, notably genes of the ETC, and genes involved in protein translation (Kim et al., 2010). The mechanism by which a mitochondrial sHSP can alter gene transcription is not clear, but is likely to result from an indirect effect and therefore probably involves other proteins and/or messengers. Mitochondria are in constant communication with the nucleus to adjust gene expression as a response to altered metabolic demand and stress (Haynes et al., 2007; Haynes and Ron, 2010; Runkel et al., 2014). Therefore, rather then initiating a signaling cascade between mitochondria and nucleus, over-expression of Hsp22 may simply modify the mitochondrial status by insuring proteostasis hence influencing mitochondrial function and integrity.

### Hsp22 Involvement in the Mitochondrial Unfolding Protein Response

Due to its drastic up-regulation upon mitochondrial protein synthesis disruption and following different types of stress, Hsp22 has been proposed to be involved in the mtUPR together with Hsp60 and mitochondrial Hsp70 (Fernandez-Ayala et al., 2010; Tower, 2014; Tower et al., 2014; Morrow and Tanguay, 2015). The mtUPR is a stress response induced by protein misfolding in the mitochondria that involves mitochondria-to-nucleus signaling (Haynes and Ron, 2010). In *Drosophila* Hsp22 has been proposed to work in an amplification loop of mtUPR since it can influence its own level of expression (Shen and Tower, 2013; Tower et al., 2014). Interestingly, the link between mtUPR and longevity is similar to the one between Hsp22 and longevity since both have to be induced before adulthood to have a positive effect (Bhole et al., 2004; Morrow et al., 2004b; Durieux et al., 2011; Houtkooper et al., 2013). Moreover, both of them have been associated with health-span in *Drosophila* (Morrow et al., 2004b; Hill and Van Remmen, 2014).

## Hsp22 Expression Reduces Mitochondrial Metabolism

Using a reporter construct consisting in the promoter of *hsp22* fused to GFP, a cell lineage-specific induction of Hsp22 was reported in oenocytes (liver-like cells) during aging (Tower et al., 2014). Interestingly, two genes were found to increase the preferential expression of the *hsp22*-GFP reporter construct during aging, namely MnSOD and Hsp22 itself (Tower et al., 2014). This link between MnSOD and Hsp22 expression was also observed in another study aimed at identifying the changes in gene expression in long-lived flies over-expressing MnSOD (Curtis et al., 2007). Oenocytes that express *hsp22*-GFP reporter were shown to accumulate less age pigment and to have lower levels of oxidative stress suggesting that Hsp22 could prevent age-induced damages by reducing mitochondrial metabolism (Tower et al., 2014). Interestingly, the reduction of the mitochondrial metabolism by Hsp22 is also supported by preliminary data from our lab that show the down-regulation of multiple isoforms of proteins from the ETC and Krebs cycle among others upon Hsp22 over-expression (Morrow et al., submitted).

## Concluding Remarks

**FIGURE 1:**
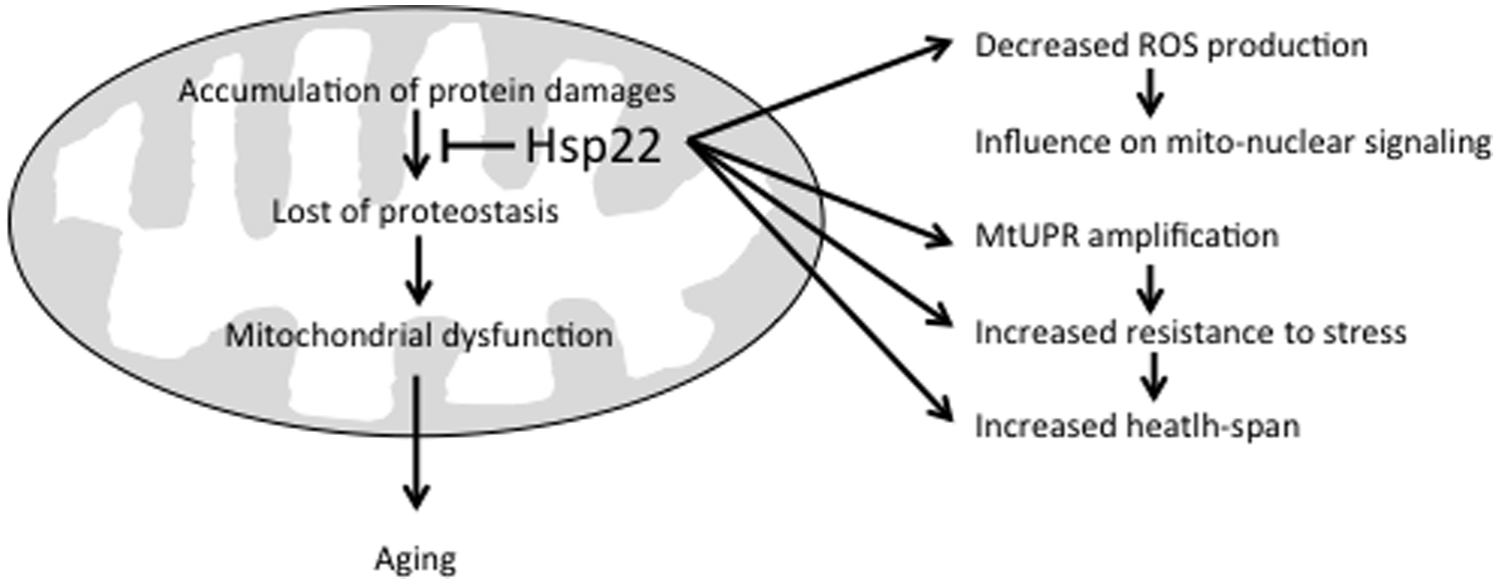
Beneficial effects of Hsp22 on mitochondria. See text for explanation.

The suggested effects of Hsp22 on mitochondria are summarized in **Figure 1**. As mentioned above, Hsp22 over-expression has been shown to increase resistance to stress and health-span (Morrow et al., 2004a,b). This can be achieved directly by preventing the accumulation of protein damages in mitochondria through its chaperone activity (Morrow et al., 2006) and/or indirectly by amplifying mtUPR signaling and subsequent expression of other chaperones and proteases (Haynes and Ron, 2010; Shen and Tower, 2013; Tower et al., 2014). The fact that a reduced mitochondrial metabolism has been associated with Hsp22 expression suggests that this sHSP may also influence mitochondria-to-nucleus signaling through a decreased ROS production (Tower, 2014; Tower et al., 2014).

*Drosophila melanogaster* Hsp22 is one of the few sHSPs found inside mitochondria independently of the cellular environment together with plants mitochondrial sHSPs (Waters, 2013) and *C. elegans* Hsp17 (Ezemaduka et al., 2014). The situation in mammals is different as all sHSPs are mainly located in the cytoplasm and shuttles to the different organelles (Nakagawa et al., 2001; van den IJssel et al., 2003; Bryantsev et al., 2007; den Engelsman et al., 2013; Marunouchi et al., 2013). This is notably the case for HSPB1, HSPB2, HSPB5, and HSPB8 that have been shown to shuttle to mitochondria in conditions of oxidative stress (Nakagawa et al., 2001; Jin et al., 2008; Marunouchi et al., 2013). As a general molecular chaperone, Hsp22 may have multiple clients inside the mitochondria and this may account for all the differences observed in flies over-expressing it as well as explain its functionality in orthologous system. Additionally to its role in mitochondrial proteostasis and mtUPR, Hsp22 could help maintain mitochondrial inner membrane integrity in a way similar to what has been observed for the mitochondrial sHSP of *C. elegans.* Indeed, when over-expressed in bacteria, ceHsp17 was shown to maintain cell envelope integrity at lethal temperatures by associating with bacterial inner membrane (Ezemaduka et al., 2014). Other sHSPs such as *Mycobacterium tuberculosis* Hsp16.3, *Synechocystis* Hsp17, and mammalian HSPB5 have been found to be associated with membranes and confer protection (Horvath et al., 1998; Cobb and Petrash, 2000; Torok et al., 2001; Zhang et al., 2005). While we have gained some important clues on the effect of Hsp22 on mitochondrial function, there is still a lot to do to understand exactly how this bona fide chaperone influences longevity and resistance to stress.

## Conflict of Interest Statement

The authors declare that the research was conducted in the absence of any commercial or financial relationships that could be construed as a potential conflict of interest.

## Conflict of Interest Statement

The authors declare that the research was conducted in the absence of any commercial or financial relationships that could be construed as a potential conflict of interest.

## ABBREVIATIONS

ETC: electron transport chain
HSE: heat shock element; HSP, heat shock protein
mtUPR: mitochondrial unfolding protein response
PQC: protein quality control
ROS: reactive oxygen species
sHSP: small heat shock protein
